# Copper(II) Binding by the Earliest Vertebrate Gonadotropin-Releasing Hormone, the Type II Isoform, Suggests an Ancient Role for the Metal

**DOI:** 10.3390/ijms21217900

**Published:** 2020-10-24

**Authors:** Lorraine Peacey, Charlotte Peacey, Adele Gutzinger, Christopher E. Jones

**Affiliations:** School of Science, Western Sydney University, Locked bag 1797, Penrith 2751, Australia; L.Peacey@westernsydney.edu.au (L.P.); charlotte.peacey@students.mq.edu.au (C.P.); 19596769@student.westernsydney.edu.au (A.G.)

**Keywords:** copper, gonadotropin-releasing hormone, neuropeptide, peptide, fertility, reproduction

## Abstract

In vertebrate reproductive biology copper can influence peptide and protein function both in the pituitary and in the gonads. In the pituitary, copper binds to the key reproductive peptides gonadotropin-releasing hormone I (GnRH-I) and neurokinin B, to modify their structure and function, and in the male gonads, copper plays a role in testosterone production, sperm morphology and, thus, fertility. In addition to GnRH-I, most vertebrates express a second isoform, GnRH-II. GnRH-II can promote testosterone release in some species and has other non-reproductive roles. The primary sequence of GnRH-II has remained largely invariant over millennia, and it is considered the ancestral GnRH peptide in vertebrates. In this work, we use a range of spectroscopic techniques to show that, like GnRH-I, GnRH-II can bind copper. Phylogenetic analysis shows that the proposed copper-binding ligands are retained in GnRH-II peptides from all vertebrates, suggesting that copper-binding is an ancient feature of GnRH peptides.

## 1. Introduction

The development of mammalian reproductive capability is dependent on successful functioning of the hypothalamic–pituitary–gonad (HPG) axis. A key peptide in the HPG axis is gonadotropin-releasing hormone I (GnRH-I), a hormone released from hypothalamic neurons into the portal circulation in the pituitary median eminence. GnRH-I travels to the anterior pituitary where it acts on gonadotrope cells to trigger the release of luteinizing hormone (LH) and follicle-stimulating hormone (FSH), which are responsible for the development of androgens in the gonads. Although GnRH-I is thought to act primarily at the gonadotrope cells, there is evidence for activity outside the pituitary, given, for example, the presence of GnRH-I receptor in the testes of many mammals [1]. GnRH-I is secreted in a pulsatile manner, and timing of the pulses is regulated by the peptides kisspeptin, dynorphin and neurokinin B [2]. Kisspeptin and neurokinin B lead to release of GnRH-I, whereas dynorphin inhibits release and therefore suppresses the GNRH-I pulse. In addition to GnRH-I, most vertebrates have a second isoform, GnRH-II, which is widely expressed in the central and peripheral nervous system, and in non-neuronal cells. This peptide is very highly conserved across all species and is thought to represent the earliest form of GnRH. The high conservation suggests that the role GnRH-II performs is critical, yet a distinct role for the peptide remains elusive and its function may differ in different species. In humans, a receptor for GnRH-II is not expressed, but the peptide can activate the GnRH-I receptor and trigger the release of LH and FSH from gonadotropes [3]. In primates, GnRH-II may have a role in regulating a pre-ovulatory LH surge [4]. In males, some species express a full-length GnRH-II receptor in the testes, and GnRH-II can trigger the release of testosterone that is independent of LH [5].

Many trace elements, including metals such as copper and zinc, have critical roles in reproductive pathways [6,7]. Copper has a long history in reproduction and has been implicated in normal physiological roles, as well as toxic processes. In the mid-1930s, Fevold et al. showed that administration of copper salts could induce ovulation in a female rabbit [8]. This effect was predominantly due to effects early in the HPG axis which trigger the enhanced release of LH [9]. The effect of copper on LH release may occur in several different ways: The metal may directly release LH by modulating the activity of GnRH receptors on gonadotropic cells [10,11]; by amplifying the GnRH-I releasing activity of prostaglandin E2 [12]; by binding to GnRH-I and enhancing or modifying its receptor-binding activity [13,14,15]; and/or by binding to neurokinin B, a peptide that indirectly modulates GnRH-I release [16,17]. The ability of copper to modify the structure and function of GnRH-I and neurokinin B and influence early events in the HPG axis has recently been reviewed [18]. Outside of the pituitary, copper can have effects directly at the gonads, and it is well documented that copper can play a role in spermatogenesis and in sperm motility [19]. Several proteins involved in copper homeostasis, including the import pump Ctr1, are expressed in germ cells and mature spermatozoa, highlighting the role copper plays in both development and then function of sperm cells [20,21,22,23]. When free, or not bound to proteins or other molecules, copper is well-known to participate in reactions that generate damaging reactive oxygen species. Indeed, high copper concentrations in the environment can have deleterious effects on sperm quality thought to be related to oxidative stress [24,25].

GnRH-I or its homologues exist throughout the Bilateria, and analysis of the evolutionary origin of GnRH and related peptides has suggested that they evolved from a common ancestor [26,27]. Recently, a GnRH-type peptide derived from the echinoderm *Asterias rubens* was shown to bind copper, suggesting that copper-binding may be a feature of the earliest forms of GnRH [28]. Although the copper-binding ability of GnRH-I is relatively well established, the ability of other isoforms has not been studied. If GnRH-II represents the earliest form of GnRH peptides, then it could reasonably be expected to bind copper if this was indeed a genuine feature of the peptide family and one that has been retained in many extant species. Thus, the objective of this study was to explore and characterize the copper-binding ability of GnRH-II.

## 2. Results and Discussion

### 2.1. GnRH-II Binds Copper with a 1:1 Stoichiometry in a Four-Nitrogen Environment

**Figure 1:**
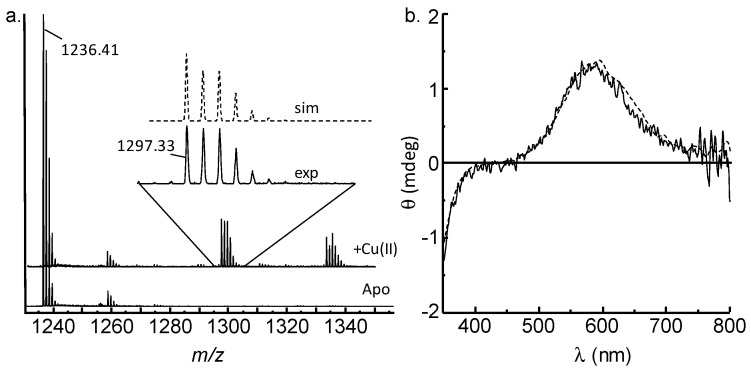
Mass spectrometry and visible-CD analysis of [Cu^II^GnRH-II]. (**a**) The mass spectrum obtained after the addition of one equivalent Cu(II) to apo-GnRH-II (100 μM) results in peaks around *m/z* 1297 that are not present in the spectrum of apo-GnRH-II. The mass and isotope distribution can be well simulated, assuming that GnRH-II loses two protons to form a mono-nuclear copper complex (*inset*). The peaks near *m/z* 1335 are an unidentified adduct of the copper complex, and the peaks at *m/z* 1236.41 are apo-GnRH-II. (**b**) The visible-CD spectrum of [Cu^II^GnRH-II] (50 μM) has a positive signal near 600 nm and a negative signal near 350 nm (*solid line*). The spectrum is identical to the visible-CD spectrum of [Cu^II^GnRH-I] (*dashed line*).

We firstly assessed the binding of copper by GnRH-II, using mass spectroscopy. The electrospray mass spectrum of GnRH-II in the absence of Cu(II) (apo-GnRH-II) shows prominent peaks due to mono and doubly charged species at *m/z* 1236.4 [M+H]^+^ (Figure 1a) and *m/z* 618.7 [M+2H]^2+^ (not shown). An additional major peak at *m/z* 1065.3 can be attributed to in-source fragmentation of GnRH-II. The addition of an equimolar amount of Cu^2+^ to GnRH-II prior to ESI-MS leads to the spectrum shown in Figure 1a (*upper spectrum*). The spectrum has predominant peaks due to apo-GnRH-II, but there is a clear singly charged peak at *m/z* 1297.33 attributable to [(M+Cu^2+^- 2H)+H^+^]^+^. At the pH (pH 7.6) of this experiment, the loss of two protons is predicted to be due to copper coordination displacing two amide protons, as is observed in many copper-binding peptides and proteins, including GnRH-I [13,28]. The peak near *m/z* 1333 is a singly charged adduct of unknown composition. Simulation of the pattern of peaks at *m/z* 1297.33, assuming a Cu^II^GnRH-II complex shows very good agreement with the experimental pattern (Figure 1b, *inset*). No peaks due to multi-copper complexes with GnRH-II were observed, despite acquiring data to 3000 Da. The presence of a peak due to [Cu^II^GnRH-II] suggests a 1:1 stoichiometry, which is the most stable stoichiometry.

We next assessed the binding, using circular dichroism in the visible wavelength region. If copper binds to a protein or peptide via amide nitrogen atoms and displaces the protons as predicted by the ESI-MS data, then quite intense peaks are generally observed in the visible region of the CD spectrum [29]. The peaks arise due to coordination-induced chirality developed around the metal binding site [30]. The visible-CD spectra obtained from [Cu^II^GnRH-II] are shown in Figure 1b (*solid line*). The spectrum has a positive signal near 600 nm and a negative signal below 350 nm that correspond to copper *d-d* transitions and most likely an imidazole → Cu(II) charge transfer transition, respectively [28,31]. Intriguingly, the position of the peaks in the CD spectrum of [Cu^II^GnRH-II] is identical to that previously obtained from [Cu^II^GnRH-I] (Figure 1b, dashed line) [32,33]. GnRH-I (pEHWSYGLRPG-NH_2_) is thought to coordinate Cu(II) via the *N*-terminal groups, incorporating deprotonated amide nitrogen atoms of the pyroglutamyl ring and His2 and the imidazole nitrogen of His2 with a solvent oxygen contributing to generate a four-coordinate copper site [13]. The CD spectra suggest that GnRH-II (pEHWSHGWYPG-NH_2_), which has the same *N*-terminal sequence as GnRH-I, is coordinating Cu(II) in a similar manner.

**Figure 2:**
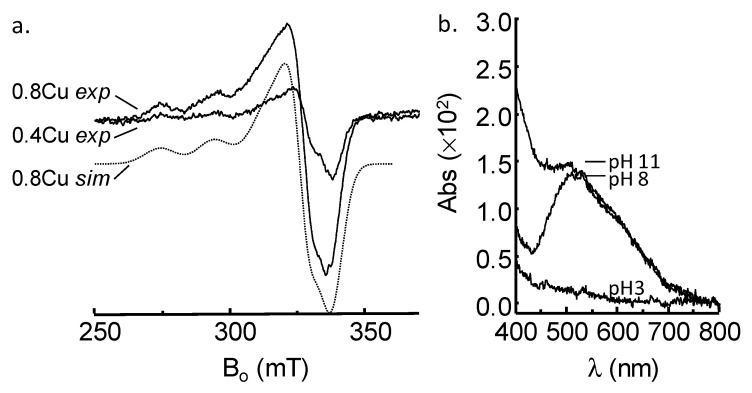
(**a**) X-band EPR spectra of GnRH-II (50 μM) in the presence of 0.4 and 0.8 equivalents of Cu(II). The dotted line, 0.8 Cu *sim*, is the simulation of the 0.8 Cu GnRH-II experimental spectrum generated by using the parameters in Table 1. (**b**) Electronic spectra of GnRH-II (100 μM) at pH 3, pH 8 and pH 11. Maximal binding occurs at pH 8, and the maximal absorption wavelength is observed at ~520 nm. A shoulder is apparent near 600 nm in both the pH 8 and pH 11 spectra.

ESI-MS and CD data suggested that amide nitrogen atoms were involved in coordinating copper, so we next used EPR spectroscopy to further investigate if nitrogen coordination was occurring. The experimental spectra derived from frozen solutions of GnRH-II in the presence of 0.2 and 0.8 equivalents of Cu(II) are shown in Figure 2a. The spectra are typical of axial-type copper complexes (g_x_, g_y_ < g_z_). The spectrum obtained from GnRH-II in the presence of 0.8 equivalents Cu(II) can be well simulated (Figure 2a, 0.8 Cu *sim*) with the parameters given in Table 1. The copper hyperfine parameters at *g_z_* were mapped to the Peisach and Blumberg plots, to establish that the copper ion in [Cu^II^GnRH-II] is most likely coordinated in an environment that contains predominantly 4N or 3N1O ligands [34]. These data are consistent with the ESI-MS and CD data. However, the hyperfine splitting at g_z_ for GnRH-II (196.8 × 10^−4^ cm^−1^) is larger than that observed for GnRH-I (176.7 × 10^−4^ cm^−1^), suggesting that the copper-GnRH-II complex contains more nitrogen donor atoms than the copper-GnRH-I complex [33]. With nitrogen ligands there is often superhyperfine coupling observable at both g_z_ and *g_x,y_* that, in many cases, can be used to estimate the number of nitrogen donor atoms [17,35,36]. However, in the case of [Cu^II^GnRH-II], superhyperfine coupling is not readily apparent, and differentiation of the spectra did not reveal additional detail, most likely due to broadening phenomena [35]. It is possible that multiple species (e.g., 4N and 3N1O species) contribute to the broadening. Analysis of the visible electronic spectrum of [Cu^II^GnRH-II] (Figure 2b) shows a λ_max_ at 520 nm at pH 8. Peaks at this wavelength are consistent with copper in a four-nitrogen site [28]. However, there is a shoulder apparent at ~600 nm, suggesting there is some copper with weaker-field donor atoms than nitrogen, most likely the inclusion of oxygen donor atoms. Indeed, GnRH-I binds copper in a site that incorporates an oxygen ligand, and the λ_max_ is near 600 nm [33]. The majority of copper is in a four-nitrogen site in GnRH-II, and there is likely a proportion that is a three-nitrogen site; however, the stoichiometry is still 1:1. In line with the EPR data, the λ_max_ at 520 nm for [Cu^II^GnRH-II] is consistent with GnRH-II having more nitrogen atoms coordinating copper than does GnRH-I. The binding is at pH 8, but a lack of coordination at pH 3 is consistent with the nitrogen donors being from an amide or the imidazole nitrogen atoms from histidine amino acids, which become available at pH values greater than the pKa of the imidazole group. GnRH-II contains histidine amino acids at positions 2 and 5, and the imidazole nitrogen of His5 is the mostly likely source of the additional nitrogen required to generate a 4N site in GnRH-II. The 4N environment suggested by the EPR and electronic spectra may seem incongruent with the CD data, which suggest that GnRH-II has an identical 3N1O site to that in GnRH-I (Figure 1b). However, at visible wavelengths, the CD spectra of copper peptide complexes are dominated by the vicinal effect arising from main chain (amide) coordination and the subsequent location of the chiral α-carbon [30]. Copper sites that incorporate only coordination by atoms from amino acid side chains have negligible visible CD spectra [37,38]. In [Cu^II^GnRH-II], the inclusion of a nitrogen from only the His5 imidazole group would not contribute to the chirality of the copper site and would be unlikely to alter the position of the main chain groups responsible for the CD signal and thus the CD spectrum retains features consistent with the site found in [Cu^II^GnRH-I]. However, the inclusion of a fourth nitrogen will affect the λ_max_ observed in the electronic spectrum (Figure 2b), due to the difference in ligand field strength, as compared to oxygen.

**Figure 3:**
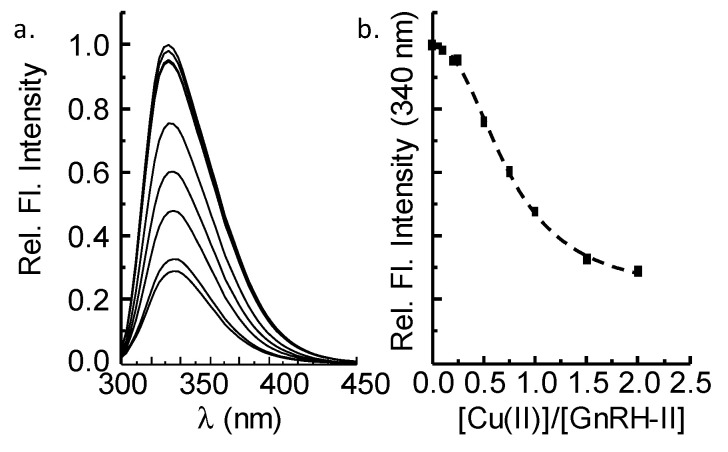
Fluorescence spectroscopy of GnRH-II. (**a**) Emission spectra of tryptophan (excitation 280 nm) amino acids in GnRH-II (50 nM) as a function of added Cu(II). (**b**) Emission at 340 nm plotted as a function of copper equivalents ([Cu(II)]/[GnRH-II]). The emission intensity has been reduced by ~50% after the addition of one equivalent Cu(II), and there is little change in intensity after 1.5 equivalents.

GnRH-II contains two tryptophan amino acids, at position 3 and 7, so we next used fluorescence spectroscopy to monitor Cu(II)-peptide binding. Excitation of apo-GnRH-II (50 nM) leads to maximal emission at ~340 nm (Figure 3a) consistent with solvent exposed tryptophan residues in a natively unstructured peptide. Titrating Cu(II) into the GnRH-II solution causes a reduction in the emission intensity, presumably due to copper–peptide interactions. At one equivalent of Cu(II), the emission has been quenched ~50%, and the addition of more Cu(II) to two equivalents causes only a further 20% quenching. In line with the MS data, this tends to suggest a stoichiometry of 1:1. Notably, the emission is not completely quenched, and we speculate that one of the two tryptophans is more affected by copper coordination than the other. We predict Trp3, adjacent to His2, a potential copper ligand, is predominantly quenched by copper coordination, whereas Trp7, more remote from the Cu(II) site, is likely to be less effected.

**Figure 4:**
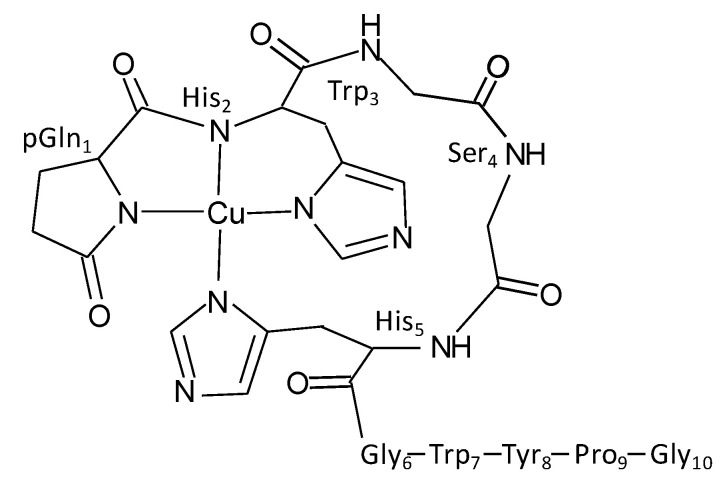
Proposed structure of the copper(II) complex of GnRH-II at pH 7.6. The side chains of Trp3 and Ser4 are omitted for clarity.

Taken together, the spectroscopic data show that GnRH-II can bind Cu(II) to form a 1:1 complex, and the EPR data and electronic spectroscopy suggest that the copper ion is in a four-nitrogen environment. GnRH-I (pEHWSYGLRPG-NH_2_) binds copper in an *N*-terminal 3N1O environment that incorporates two amide nitrogens and an imidazole nitrogen (i.e., {2N_a_, N_im_, O}), and GnRH-II (pEHWSHGWYPG-NH_2_) contains the same *N*-terminal sequence and only differs from GnRH-I by three amino acids [13,33]. Notably, GnRH-II includes a second histidine at position 5, and the spectroscopic data are consistent with GnRH-II binding copper via the *N*-terminal pEHxxH sequence in a coordination sphere that includes two amide and two imidazole nitrogens (i.e., {2N_a_, 2N_im_}) to generate the four-nitrogen environment (Figure 4). The inclusion of a ‘remote’ imidazole ligand is not uncommon, and, indeed, the proposed [Cu^II^GnRH-II] site has some similarity to the copper site in a peptide fragment of human angiogenin [31].

### 2.2. Histidine Amino-Acids Are Highly Conserved in Vertebrate GnRH-II

**Figure 5:**
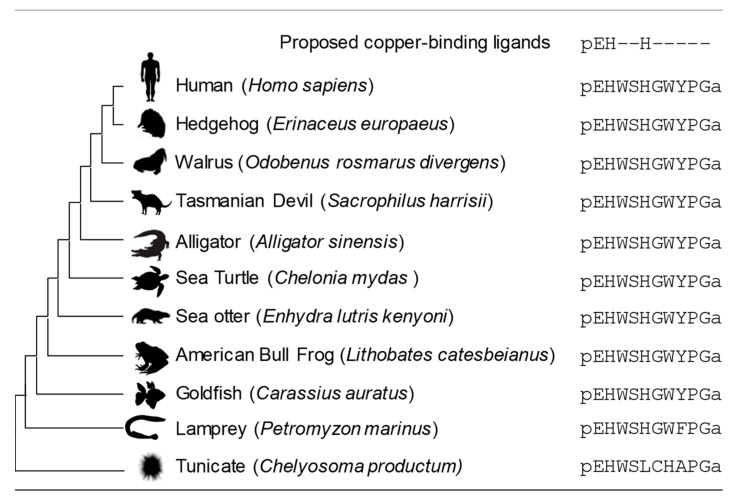
Diagram showing GnRH-II sequences from deuterostomes. All vertebrate GnRH-II peptides contain the pEHxxH sequence proposed to be the copper binding site. The site is invariant in vertebrates and is not observed in deuterostome invertebrates, such as the tunicates. The peptides are all amidated at the *C*-terminus, denoted by an ‘a’ in the primary sequence.

The sea lamprey *Petromyzon marinus* is considered a basal vertebrate, having remained unchanged for millennia. Lamprey GnRH-II contains the pEHxxH sequence containing the proposed copper ligands, suggesting that the ability to coordinate copper was a feature of the earliest vertebrate peptide. The primary sequence of GnRH-II has remained largely invariant for several million years of vertebrate development, suggesting strong selective pressure to retain the sequence (Figure 5) [27,39,40,41]. The retention of the copper ligands suggests the metal may influence GnRH-II structure and function in all vertebrate species. The invertebrate chordates tend to not contain a peptide with a pEHxxH sequence despite expressing several GnRH-I isoforms [27,42]. However, the tunicate *Chelyosoma productum* has a peptide identified as a tGnRH-II that contains a pEHxxxxH sequence (Figure 5) [43]. Although the presence of this sequence implies the peptide may bind metal with some similarity to vertebrate GnRH-II, we predict it is unlikely given tGNRH-II forms a disulfide-linked homodimer that would sterically hinder His7 from participating in metal coordination [43,44].

## 3. Materials and Methods

### 3.1. Materials

Mammalian gonadotropin-releasing hormone II (GnRH-II, pEHWSHGWYPG-NH_2_) was synthesized by Synpeptide (Shanghai, China), at > 95% purity, and was not purified further. The concentration of this peptide was determined by using the extinction coefficient of tryptophan (5690 M^−1^ cm^−1^) and tyrosine (1290 M^−1^ cm^−1^) at 280 nm. A stock solution of CuCl_2_·H_2_O was prepared in Milli-Q water (18 MΩ, MerckMillipore, Victoria, Australia) that was metal-free, as determined by electron paramagnetic resonance. Working solutions of CuCl_2_·H_2_O were prepared from the stock solution on the day of use. The buffer was 20 mM 4-ethylmorpholine (Sigma-Aldrich, Castle Hill, Australia), pH 7.6 unless otherwise indicated. This buffer does not coordinate copper.

### 3.2. Mass Spectroscopy (MS)

Mass spectroscopic data were acquired on a XEVO-QToF instrument controlled by MassLynx software (Waters Corp., Milford, MA, USA). Spectra were collected in positive ion mode, the capillary voltage was 3.5 V, the cone voltage was 30 V, the source temperature was 80 °C and the mass range was 200–3000 Da. Peptide samples were diluted, using a 1:1 acetonitrile:water solution containing 0.1% formic acid. The samples were applied to the interface by direct infusion, at a flow rate of ca. 10 μL/min. Masses were calibrated to leucine enkephlin *m/z* 556.28. Spectra were simulated by using IsoPro3.

### 3.3. Electronic Spectroscopy

UV/Visible spectra were acquired on a Cary 100 spectrometer operated with WinUV software (Agilent Technologies Australia, Victoria, Australia). All spectra were collected by using a 1.0 cm pathlength cuvette over a wavelength range of 200–800 nm. The scan rate was 300 nm/min.

### 3.4. Fluorescence Spectroscopy

Fluorescence spectra were acquired using an FS5 fluorimeter (Edinburgh Instruments Ltd., Livingston, Scotland, UK). A 1.0 cm × 1.0 cm far-UV quartz cuvette (Spectrocell, Oreland, PA, USA) was used for all measurements. Tryptophan emission was collected over the range 300 nm – 450 nm after excitation at 280 nm (5 nm slit width). Spectra were collected at room temperature (~25 °C).

### 3.5. Electron Paramagnetic Resonance (EPR)

X-band (~9.4 GHz) EPR spectra were collected on a Bruker EMXplus spectrometer equipped with an EMX PremiumX bridge (Bruker BioSpin, Victoria, Australia). The temperature was held at 150 ± 5 K, using nitrogen vapor regulated by a ER4131 variable temperature controller. The power was 0.63 mW, the modulation frequency was 100 kHz and the modulation amplitude was 5.0 G. Twenty scans were acquired and averaged for each sample, and a baseline (buffer-only) spectrum was subtracted from each sample spectrum. Simulation of the EPR spectra was performed by using Easyspin running in MatLab 2020a [45]. Simulations used matrix diagonalization to determine the copper hyperfine and Zeeman parameters, and linewidths were fitted by using a correlated distribution of *g*- and *A*-strains [46].

### 3.6. Circular Dichroism (CD) Spectroscopy

CD spectra were acquired on a Jasco J810 spectrometer at room temperature (~23 °C) (Jasco, Easton, MD, USA). Spectra were collected from 300 to 800 nm, data were acquired every 2 nm and at least 10 scans were averaged. A 1.0 cm pathlength cuvette was used.

### 3.7. Phylogenetic Analysis

The human GnRH-II sequence was used as a search sequence for BLAST analysis in Uniprot. A selection of vertebrate and invertebrates were chosen for comparison. Peptide sequences were also obtained from the published literature [26]. MEGAX (Molecular Evolutionary Genetics Analysis across computing platforms) and ClustalW were used to align sequences [47].

## 4. Conclusions

GnRH-II is widely distributed in the central and peripheral nervous system and in non-nervous tissues, including reproductive tissue. The primary sequence has remained unchanged for 500 million years, suggesting that the peptide has a role important enough to drive evolutionary pressure to retain the sequence; however, that function remains unclear. The work described here shows that GnRH-II binds copper in a site predicted to include amide nitrogen and histidine imidazole nitrogen donor atoms derived from the pEHxxH *N*-terminal sequence. GnRH-I binds copper via the pEH sequence (i.e., {2N^−^, N_im_}) with a solvent derived oxygen as the fourth donor atom [13]. The data presented here predict that GnRH-II binds similarly to GnRH-I, but instead of a solvent oxygen, the imidazole of His5 is the fourth ligand. The binding of the His5 side chain will require movement of the backbone of GnRH-II, to generate the four-coordinate geometry (Figure 4). We speculate that the copper-induced structuring of GnRH-II will modify how the peptide interacts with a receptor, possibly the GnRH-I receptor in humans. The binding of copper may also allow GnRH-II to interact with a different receptor, consequently leading to functional diversity, depending on copper availability in the tissues where GnRH-II is expressed. Further work investigating the physiological consequences of copper-coordination remains to be explored. The binding of copper by GnRH-II and GnRH-I suggests that metal coordination has been a feature present in the earliest form of the peptide and has been retained during gene-duplication events that gave rise to other GnRH isoforms [41]. The reasons for the pressure to retain copper-binding ability in all deuterostome GnRH isoforms are unknown, but the work described here opens bioinorganic avenues of investigation that may lead to a greater understanding of GnRH-II function in vertebrate biology.

## Figures and Tables

**Table 1:** Simulation parameters for the EPR spectrum of [Cu^II^GnRH-II].

Parameter	Value
*g_i_* (*i* = x, y, z)	2.067, 2.067, 2.222
*A_i_* (^63^Cu) (×10^−4^ cm^−1^)	10.0, 10.0, 196.8
Linewidth (×10^−4^ cm^−1^)	4.67
*g*-strain*_i_*	0.052, 0.058, 0.086
*A-*strain*_i_* (×10^−4^ cm^−1^)	11.7, 11.7, 3.3
