# Correction: Peacey, L., et al. Copper(II) Binding by the Earliest Vertebrate Gonadotropin-Releasing Hormone, the Type II Isoform, Suggests an Ancient Role for the Metal. *Int. J. Mol. Sci.* 2020, *21*, 7900

**DOI:** 10.3390/ijms22073431

**Published:** 2021-03-26

**Authors:** Lorraine Peacey, Charlotte Peacey, Adele Gutzinger, Christopher E. Jones

**Affiliations:** School of Science, Western Sydney University, Locked bag 1797, Penrith 2751, Australia; L.Peacey@westernsydney.edu.au (L.P.); charlotte.peacey@students.mq.edu.au (C.P.); 19596769@student.westernsydney.edu.au (A.G.)

The authors wish to make the following correction to this paper [1]. Due to an incorrect phylogenetic tree, Figure 5 should be replaced with the following figure (Figure 1).

The correction does not change the scientific conclusions of the article in any way.

The authors would like to apologize for any inconvenience caused to the readers by these changes.

## Figures and Tables

**Figure 1:**
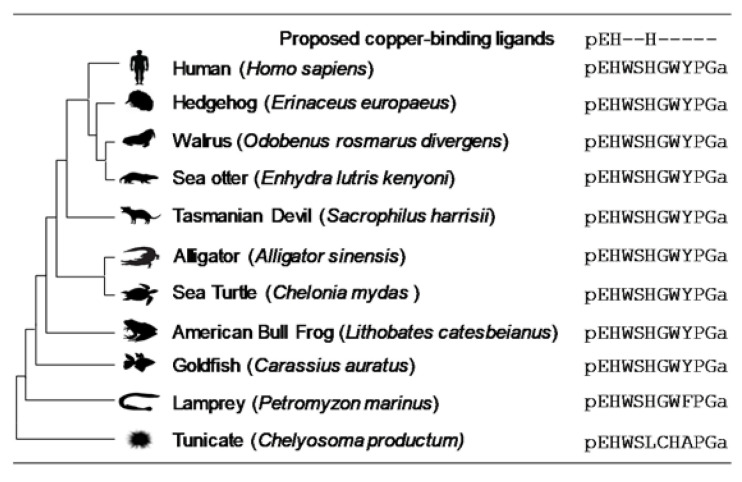
Diagram showing GnRH-II sequences from deuterostomes. All vertebrate GnRH-II peptides contain the pEHxxH sequence proposed to be the copper-binding site. The site is invariant in vertebrates and is not observed in deuterostome invertebrates such as the tunicates. The peptides are all amidated at the C-terminus, denoted by an ‘a’ in the primary sequence.
